# Extravasation of Urine Followed by Extensive Sloughing of the Scrotum, Exposing Both the Testicles, Attended with Severe Constitutional Symptoms, and Terminating Fatally

**Published:** 1827-09

**Authors:** 

**Affiliations:** St. Thomas's Hospital.


Case II. *Extravasation of Urine followed by extensive Sloughing of the Scrotum, exposing both the Testicles, attended with severe Constitutional Symptoms, and terminating fatally.* Treated by Mr. Green, at St. Thomas's Hospital.

September 14th, 1826.—Philip Tame, æt. fifty, of thin and spare habit, and regular and abstemious in diet, perceived, about six or seven weeks ago, a hard and painful swelling on the right side of the anus, which, after applying several leeches and poultices for a few days, broke, and discharged a quantity of pus. He has continued to apply poultices, and there now remains a sinus, undermining the surrounding integument to a small extent, with scarcely any thickening or induration. The abscess appears to have been superficial, as, after an attentive examination, no deep sinus can be traced.

Dilate and poultice.

16th.—The wound is indolent, and the patient's system much depressed; pulse frequent, small, and feeble; tongue foul; appetite bad.

℞. Dec. c. Tr. Cinchonæ ter die sumend.—Cerev. ℔j. indies.—Catap. commune.

20th.—Is rather better: the wound has a more healthy appearance, and is beginning to granulate; the bowels are confined.

℞. Submur. Hyd. gr. v.; Pulv. Scammon. gr. x. M. statim sumend.

23d.—Has had a severe rigor; complains of weakness, and the general health is evidently much disordered. Pulse small, quick, and fluttering; tongue white; countenance pallid and anxious.

25th.—Has had a repetition of the rigors; there is some swelling, induration, and tenderness of the perineum.

Hirud. x. perinæo applicendi, et postea Catap. Lini.

26th.—The scrotum and perineum are much swollen, discoloured, and vesicated; the urine is retained, and there is some swelling and tenderness above the pubes. The patient is very low, and slightly delirious; countenance extremely anxious; pulse quick and small; tongue rather brown and dry; great thirst. Under these circumstances, in the absence of Mr. Travers, under whose care the patient was first admitted, Mr. Green made an incision through the raphè of the scrotum and perineum, giving exit to a considerable quantity of urine, mixed with fetid, ill-conditioned pus. A catheter was then introduced into the bladder, and confined in that situation by a T bandage, and a large poultice applied over the injured parts.

27th.—Has passed a restless night, notwithstanding great drowsiness; the pulse is extremely small and feeble; nearly the whole scrotum is in a state of gangrene.

℞. Tr. Opii m. v.; Ammon. Carb. gr. v. ex Mist. Camph. sextâ quâque horâ sumend.—Porter ℔j. quotidie.—Sago, Syrup, Port wine ℥ ij. indies.—Catap. commune.

30th.—Continues very low and weak. The gangrene has not extended, but there does not appear to be any disposition to throw off the slough.

Porter ℔ij. quotidie.—Catap. Cerev.

October 2d.—The slough has partially separated from the scrotum, exposing both testicles; the surface is pale and smooth, without the least attempt at granulation. The urine passes partly through the catheter, and partly through the wound in the perineum; the bowels are much relaxed.

Omit the Porter, and substitute Port-wine ℥ iv. Brandy ℥ ij. indies.

4th.—The bowels continue much relaxed. The discharge from the wound is copious, and very thin; the slough has completely separated, but there is not the slightest appearance of any attempt at granulation. The patient is very drowsy, but is on the whole rather better.

℞. Conf. Aromat. ℈j.; Ammon. Carbon. gr. x.; Tr. Opii m. x. ex Mist. Camphor. sextâ quâque horâ sumend.

8th.—The last two days he has become much more feeble; the drowsiness has increased, and low muttering delirium is frequently present. The pulse is extremely small and fluttering; the tongue coated with a thick brown fur, but is moist; the feces escape involuntarily.

Brandy ℥ vj. quotidie.

During the following day he gradually sunk, and on the morning of the 10th he expired.

*Post-mortem examination.*—The inferior portion of the urethra was destroyed to a considerable extent. In endeavouring to remove the contents of the pelvis, the cavity of a large abscess was opened, which extended from the opening at the verge of the anus, between the bladder and rectum, as high as the level of the first bone of the sacrum, but did not communicate with the bowel or with the bladder, which were both healthy. The structure of the viscera presented no morbid appearance.

